# Who Called Team Europe? The European Union’s Development Policy Response During the First Wave of COVID-19

**DOI:** 10.1057/s41287-021-00428-7

**Published:** 2021-07-12

**Authors:** Aline Burni, Benedikt Erforth, Ina Friesen, Christine Hackenesch, Maximilian Hoegl, Niels Keijzer

**Affiliations:** Deutsches Institut Für Entwicklungspolitik, Tulpenfeld 6, 53113 Bonn, Germany

**Keywords:** European Union, Foreign aid, Development policy, Norm diffusion, COVID-19

## Abstract

The COVID-19 pandemic is a critical juncture for global development. Under the label of “Team Europe”, the EU has sought to mobilize rapid development assistance to support partners in addressing the impacts of the crisis, while promoting joined-up approaches among European actors to assert itself in a changing and competitive geopolitical context. This article assesses how substantive and process-oriented EU development policy norms are reflected in the Union’s global COVID-19 response. Focusing on the EU’s response during the first wave of the COVID-19 pandemic in the first half of 2020, the article shows that the EU’s response to this extraordinary crisis consisted of a deepening of EU integration. In so doing, the EU emphasized process-oriented over substantive norms in its development policy.

## Résumé

La pandémie de COVID-19 représente un moment de transition critique pour le développement international. En utilisant l’étiquette « Équipe Europe », l'UE a cherché à mobiliser une aide au développement rapide pour aider ses partenaires à faire face aux impacts de la crise, tout en favorisant des approches conjointes entre les acteurs européens pour s'affirmer dans un contexte géopolitique changeant et concurrentiel. Cet article évalue la façon dont les normes de la politique de développement de l'UE, qui portent sur le fond et sont axées sur les processus, se reflètent dans la riposte de l'Union à la COVID-19 à l’échelle mondiale. En se concentrant sur la réponse de l'UE lors de la première vague de pandémie de COVID-19 durant le premier semestre 2020, l'article démontre que la riposte de l'UE à cette crise sans précédent a consisté en un renforcement de l'intégration européenne. Ce faisant, dans sa politique de développement, l'UE a mis l'accent sur les normes axées sur les processus plutôt que sur les normes qui portent sur le fond.

## Introduction

Having evolved from a global health crisis into a broader social, economic, and political crisis, the COVID-19 pandemic is a critical juncture for global development. A combination of isolationism, scapegoating, “politics of generosity”, mask diplomacy, as well as disinformation has shown that the initial handling of the pandemic reinforced geopolitical competition rather than promoting international cooperation. The pandemic has accelerated existing trends in geopolitics, such as the shift of power and influence away from Europe and the United States to Asia, specifically China. The pandemic has also raised important questions on the ability of multilateral institutions to effectively contribute to collective problem-solving. In this challenging global context—and facing criticism for its inefficient early response by worst-hit member states such as Italy and Spain—the European Union (EU) sought to position itself as a community of values, proclaiming its unity and power in the spirit of a global Team Europe reaction to the pandemic (EU, 2020b).

The EU is frequently depicted as a normative power (Manners, 2002) that shapes the world order by promoting free markets, welfare systems, and a rules-based approach to global governance. The “normative power Europe” concept considers the EU an “ideational” actor in global politics and focuses on how it promotes common principles and acts to diffuse norms (Whitman, 2011). Such norms, or standards, have been defined as a “shared expectation by a community of agents for appropriate behaviour” (Finnemore, 1996:156). The EU’s development policy plays an important and distinct role in the promotion of European values and is guided by the Union’s self-understanding as an ardent defendant of a rules-based multilateral global order and a community of Western liberal values. A key component of the EU’s action on the international scene is development policy, which the EU treaties stipulate “shall be guided by the principles which have inspired its own creation, development and enlargement” (EU, 2012: Art. 21). In the context of this article, the norms the EU is promoting are considered to be either substantive (“what”) and include the promotion of sustainable peace, freedom, democracy, human rights, rule of law, equality, social solidarity, sustainable development, and good governance, or they are considered to be process-oriented (“how”), which includes the commitment to multilateralism. The EU articulates and diffuses these norms in biannual meetings of the EU’s development ministers, official statements, international negotiations, or other global processes.

In the early 2000s, EU development policy constituted an independent and self-standing policy area that was associated with overall increasing aid budgets and a focus on human development and poverty reduction in the context of the Millennium Development Goals. The EU actively promoted the international aid effectiveness agenda and sought to offer a coherent approach to development policy by bundling national and supranational policies and actions under the common umbrellas of the European Consensus on Development (2005), the division of labour initiative (2007), and joint programming (Carbone, 2013, 2017; Delputte and Orbie, 2014).

However, the 2008 global financial and economic crisis and the increasing numbers of refugees entering the EU in 2015/6 changed the EU’s approach to development policy. Rather than focusing on poverty eradication—as is its overall objective according to the Lisbon Treaty (Art. 208 TFEU)—development policy has been gradually instrumentalized for other policy objectives related to trade and investment promotion, security, and migration (Bergmann et al, 2019). This instrumentalization has happened in a period during which the EU’s development policy debate has been characterized by increasing levels of politicization (Hackenesch et al, 2021). In light of instrumental tendencies of EU development policy coming to the fore in times of crises, this article explores whether similar patterns can be observed in EU development policy debates concerning the response to the COVID-19 pandemic.

On 30 January 2020, the World Health Organization (WHO) declared the first outbreak of a novel coronavirus, sparking a public health emergency of international concern. The EU had already activated its integrated political crisis response mechanism on 28 January 2020 in order to assist EU citizens in Wuhan, China. Despite the EU having limited legal competencies in the area of health policy, it has continued to closely monitor the situation and has introduced various initiatives, including the adoption of relevant EU legislation and coordinating with member states to share information, assess needs, and ensure a coherent EU-wide response (Goniewicz et al, 2020). In early April 2020, the EU presented its global response to the pandemic as a “Team Europe” approach—a joined-up strategy based on joint priorities, a combined financial package, support for global preparedness, and the promotion of global coordination and multilateralism (EU, 2020b).

The EU’s global response to the COVID-19 pandemic and the Team Europe approach constitute a test case of the EU’s efforts to diffuse its norms and policies abroad in a changing geopolitical context. Against this backdrop, this article analyzes the EU’s external response during the first wave of the COVID-19 pandemic in the first half of 2020, with a specific focus on the construction of the idea of Team Europe. Its central argument is that the main motivation for the Team Europe package was the strengthening of European integration in development policy and the desire to augment Europe’s profile and collective visibility as a development cooperation actor. In doing so, the EU has clearly emphasized process-oriented norms over substantive development policy norms. The efforts to make EU support more “visible” and to strategically communicate the EU’s global COVID-19 response can, in part, also be viewed as a response to China’s increased power projection from the outset of the crisis.

The article is based on a structured review of existing research on norms and EU development policy as well as an analysis of EU policy proposals and public statements related to the COVID-19 pandemic from March to July 2020. The analysis of the EU’s policy response, which is published as an appendix along with this paper, was complemented by the authors’ direct observations during a series of online seminars organized by think tanks to discuss and consider the EU’s global COVID-19 response (see Annex Table 2).

In the next section, we present the conceptual framework, which considers how norms are produced and featured as well as how they have been articulated in the EU’s development policy since the early 2000s. This discussion provides the analytical lens for the subsequent analysis of EU policy proposals during the early stages of the pandemic. Following the conceptual framework, we present a narrative of the events related to the construction of the EU’s external response, and more specifically its Team Europe approach. We also examine in more detail key policy documents and analyze how the EU presents substantive and process-oriented norms in its global COVID-19 response. A final section presents overall conclusions and avenues for further research.

## Norm articulation and diffusion in EU development policy

International Relations research considers norms to be a key explanatory factor for influence in world politics. In this context, the EU is regarded as an unusual case by being neither a state nor a multilateral organization but a norm community consisting of “actors that share expectations about appropriate behaviour as well as norms that define this understanding of ‘appropriateness’” (Björkdahl, 2012:83; see also Finnemore, 1996). Manners, most prominently, claims that the EU is a “normative power” which differs from other global actors because it acts in a normative way and promotes normative principles that, within the United Nations (UN) system, are acknowledged to be universally applicable (Manners, 2002, 2008, 2009). Whitman (2011) similarly refers to the EU as an “ideational” actor in the field of foreign policy. The Treaty on European Union (TEU) specifies that the values and principles which inspired the founding of the European project—sustainable peace, freedom, democracy, human rights, rule of law, equality, social solidarity, sustainable development, and good governance—should also guide its external action (Art. 2 TEU and Art. 3.5 TEU). In addition to these substantive norms (the “what” of cooperation), the TEU also defines process-oriented norms that refer to the “how” of cooperation and includes the commitment to “promote multilateral solutions to common problems, in particular in the framework of the United Nations” (Art. 21 TEU).

The Lisbon Treaty defines development policy as a shared competence between the EU and the member states, and identifies “the reduction of poverty, and in the long term, the eradication of poverty” as its overall objective (Art. 208 TFEU). In 1992, the Maastricht Treaty had first introduced the EU’s legal basis for development policy and specified three key process-oriented principles to ensure collective effectiveness: coherence, complementarity, and coordination (Hoebink, 2004). Although the EU and its member states retain independent (bilateral) development policies, these three principles serve to further promote integration in this policy area and contribute to the EU’s collective effectiveness and legitimacy. In this context, the EU has three roles in development: It is a donor in its own right, it acts as a coordinator of European development issues, and it aspires to be a norm-maker on the international development scene (Orbie, 2012:22).

In the early 2000s, the EU put a strong emphasis on poverty reduction and human development to align its development policy with the Millennium Development Goals. The objective to increase official development assistance (ODA) provided by the EU and member states to comply with the internationally agreed 0.7 target also played a strong role in European development policy debates (Orbie, 2012:23; Bergmann et al, 2019).

Although these norms remain at the centre of the EU’s development policy and are reaffirmed and further developed by the 2030 Agenda for Sustainable Development and its 17 Sustainable Development Goals, the increased geopolitical competition—particularly in Africa—has prompted the EU to pursue a more pragmatic and interest-driven approach (Grimm and Hackenesch, 2017). Particularly during and since 2015, the EU’s overall external policy has increasingly used the EU’s development policy and its considerable budget to advance objectives in areas such as migration, security, and investments. Partially driven by the pragmatism and assertiveness which have characterized the EU’s external policy since 2015, most of the key EU development policy norms have been articulated in a “crisis-response” mode. The EU’s “neighbourhood” and the African continent have been viewed increasingly as loci of emerging crises. Since 2015/16, in the wake of a high influx of migrants, EU development cooperation with these countries has increasingly emphasized efforts to address assumed “root causes” of migration and a related focus on job creation (see also Hackenesch et al, 2021). In doing so, the EU has moved away from emphasizing developing-country benefits as the aim of development cooperation towards the pursuit of “mutual benefit”, presenting its self-interest as a donor and development cooperation recipients’ needs as two legitimate and simultaneously attainable goals of development cooperation (Keijzer and Lundsgaarde, 2018; Mawdsley, 2018). More recently, the EU has turned towards a narrative grounded in the idea that the block needs to be more “geopolitical” with the von der Leyen Commission, which has been in office since 2019 and has promised to be a “geopolitical Commission”.

The rapidly developing coronavirus outbreak challenged the EU’s crisis management capabilities within and beyond its borders. As a reaction to the initial shock and facing growing criticism—in part even propelled by Chinese defamation efforts targeted at the EU (Small, 2020)—the EU was quick to propose a common European response to COVID-19. The next section identifies how key development policy norms were articulated (or de-emphasized) in the EU’s proposed global response under the heading of Team Europe.

## The EU’s global response to COVID-19: Team Europe

**Fig. 1:**
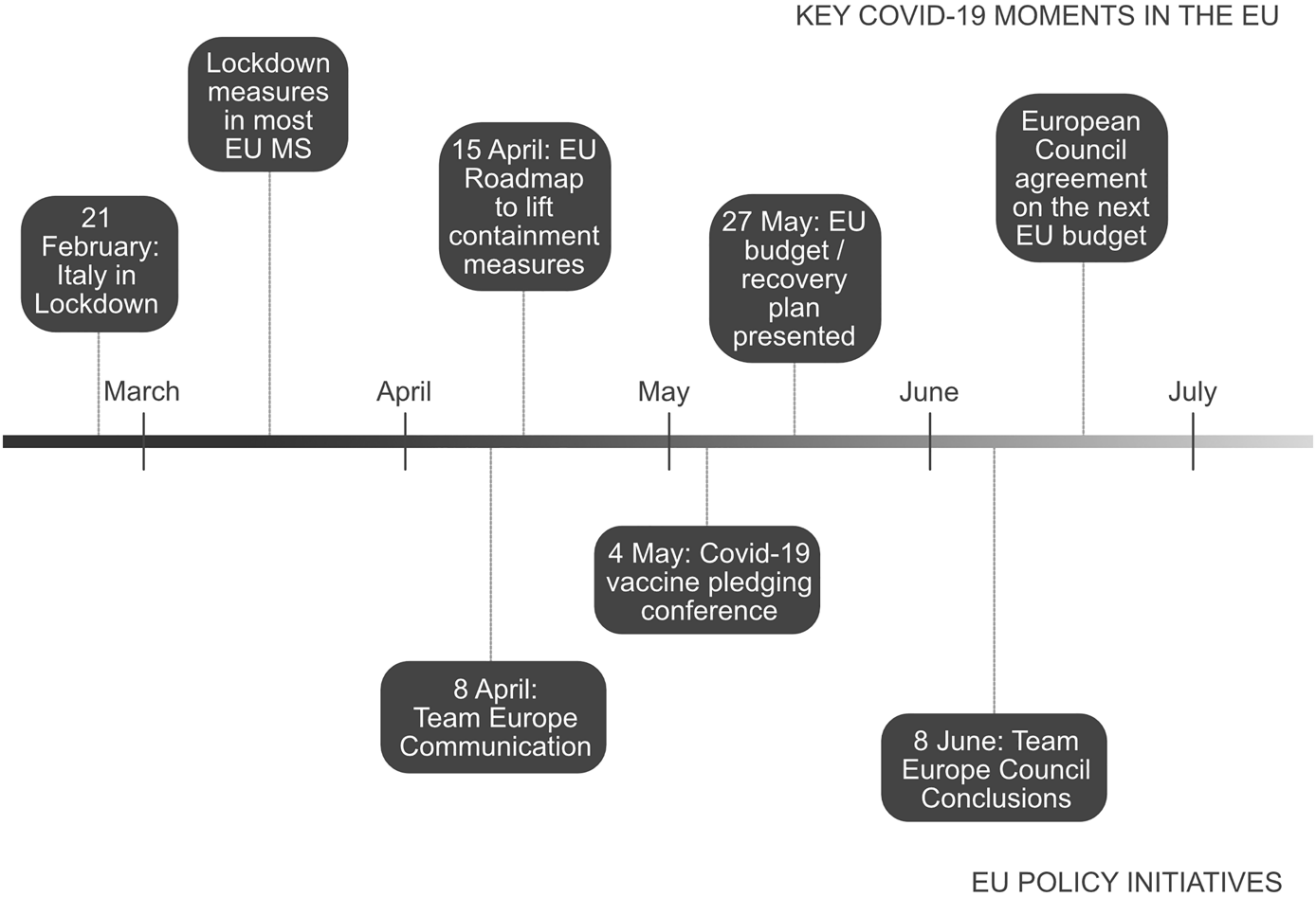
EU COVID-19 global response timeline

Since the start of the global pandemic, the EU has used various forms of written and verbal communication to present its proposed global response (see Annex Table 3). The EU’s global response was first presented on 8 April by means of a Joint Communication by the European Commission and the European External Action Service (EU, 2020b)^1^ that was prepared for an informal meeting of EU development ministers the same day (see Fig. 1 for an overview of key events and EU policy documents in the first half of 2020). Reiterating its position as the world’s largest donor and a leading economic power at the forefront of the global effort to respond to the pandemic, the EU called for a “fast, massive and coordinated global response” (EU, 2020b:1). The EU labelled its external COVID-19 response as the “Team Europe” approach—a single framework of action for European external responses that combines contributions from all EU institutions, EU member states, and EU financial institutions in order to provide “a critical mass that few others can match” (EU, 2020b:1). The Joint Communication proposed that Team Europe would be a joined-up strategy based on four main pillars: joint priorities, a joint financial package, support for global preparedness, and the promotion of global coordination and multilateralism. The Communication further stressed that any European response should be guided by core values (good governance, human rights, the rule of law, gender equality and non-discrimination, decent work conditions, and humanitarian principles) and strategic interests (EU, 2020b:2).

This commitment to finding a global solution to the pandemic was followed by a high-profile pledging conference on 4 May, which aimed at raising funds to accelerate the development of a vaccine that was “universal and affordable” (EU, 2020b). Following the first ministerial exchange on 8 April, EU ministers responsible for development cooperation reconvened in the Foreign Affairs Council on 8 June to adopt Council Conclusions on Team Europe, which presented a political statement (EU, 2020c) and were accompanied by an updated table with the financial contributions made by all associated EU actors.^2^

Two key priorities of the EU’s Team Europe response can be derived from the press releases and political statements that were issued between March and July 2020 (see Annex 3): (1) pursuing a common European approach, and (2) supporting multilateral solutions. Building on the above discussion of norms articulation, Table 1 illustrates how the EU’s substantive and process-oriented development policy norms could inform these two priorities.

**Table 1:** Substantive and process-oriented EU development policy norms according to the EU’s Lisbon Treaty and relevant development policy strategies. Source: Own elaboration

Examples of substantive EU norms (“What?”)	Examples of process-oriented norms (“How?”)
Poverty reduction as the overall objective of EU development policy Fundamental EU values to be promoted through its external action (EU Charter of Fundamental rights; Convention on Human Rights) Policy commitments (e.g. SDGs)	Coordination, complementarity, and coherence of EU development policy Working better together agenda (also referred to as “joint programming”) Commitment to aid effectiveness principles and to advancing multilateral solutions

In the following, we further describe the EU’s proposals to pursue both priorities. For each in turn, we analyze how they are presented in the relevant policy documents and how substantive and process-oriented norms have shaped them. The analysis is based on a qualitative text analysis of the key policy documents, press releases, and other communication outputs listed in Annex 3 (see data appendix for full analysis). Additional observations were derived from participant observations in several online meetings with European decision-makers, civil society actors, and academics discussing the proposed measures (see Annex 2).

## First priority: The pursuit of a common European approach to development cooperation

In the initial months following the outbreak of the COVID-19 pandemic, the EU’s global response was characterized by a focus on collective action and process-oriented development policy norms. However, as agreed in the ministerial statement of 8 June (EU, 2020c) and guided by the proposals of the Commission and the External Action Service of 8 April (EU, 2020b), the EU’s response also emphasized several substantive norms.

In terms of substantive norms, the 8 April Communication frames a strong global European response as a “matter of upholding our core values” whereby the “wellbeing of our partners across the globe matters to every European” (EU, 2020b:1). Moreover, the EU expressed an eagerness to show its solidarity with affected vulnerable populations across the rest of the world and to contribute towards immediate short-term crisis management in the health sector. Other substantive EU development policy norms such as human rights, the rule of law, good governance, gender equality, democracy and fundamental values, and humanitarian principles did not feature prominently in the policy documents in the first half of 2020 and were referred to twice by the Commission (EU, 2020b) and once in the ministerial statement (EU, 2020c).

In spring 2020 there were high levels of uncertainty about how the pandemic would evolve and a strong focus on supporting the short-term challenges encountered by developing countries in their health sectors. With regards to the long-term recovery, the Commission notes on one occasion that the “global response to COVID-19 will integrate the strategic objectives the EU has set itself as regards the environment and climate” (EU, 2020b). The Council (EU, 2020c) aims “to build back better” and at “promoting equitable, sustainable and inclusive recovery processes in line with the proposed European Green Deal”.

**Fig. 2:**
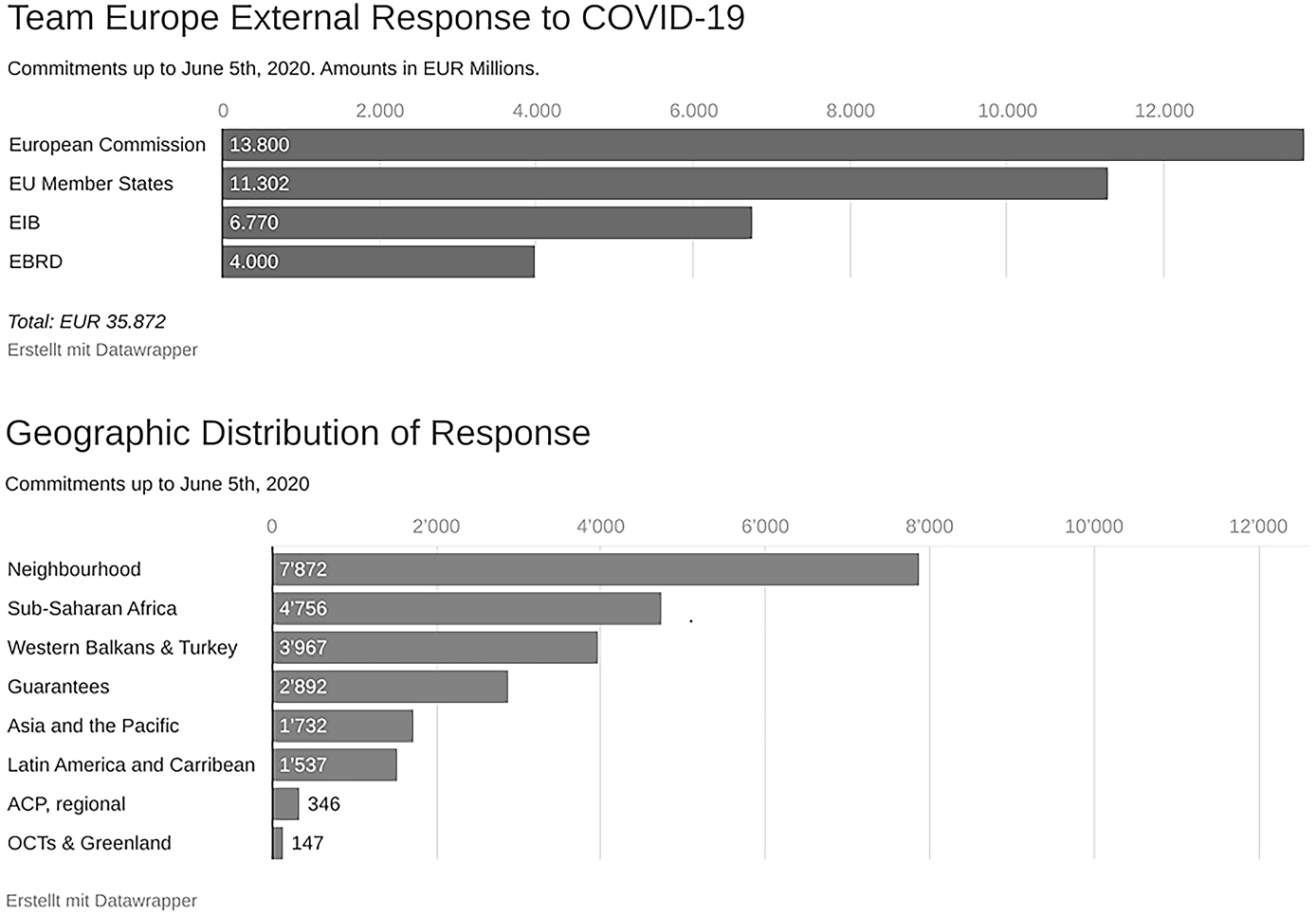
Team Europe external response

In terms of process-oriented norms, the EU’s response emphasized the mobilization of funds, specified the amounts needed, and outlined how these funds should be provided. The strong emphasis on the total volume that the EU was willing to spend seemed to also be driven by the objective to increase the visibility of EU action. This showcasing of the magnitude of the EU’s support in monetary terms was at least in part motivated by the perceived need to counterbalance China’s mask diplomacy. As the EU was nearing the end of its seven-year budgetary cycle, it was particularly challenging for it to provide a financial response to the pandemic since all funds from the current budget had already been committed and the EU was unable to mobilize additional funds. As a result, the EU had to rely on available reserves and reallocate funds to the new priorities (Jones et al., 2020). The financial resources initially dedicated to the COVID-19 response—a mix of grants, loans, and guarantees—were first communicated on 8 April in the form of a table with the financial contributions made by all associated EU actors, and then in amended form in June (Fig. 2).

The political agreement by EU heads of state and government on 21 July on the “Next Generation EU” (NGEU) package along with the normal seven-year budget has been described as historic. Yet, the budgetary resources for international cooperation, development, and cooperation with the neighbourhood were capped at €70.8 billion, 10 per cent less than what the Commission had originally proposed in May 2018 because the NGEU package did not contain funds for external action. Two days after the European Council meeting, the European Parliament adopted a resolution that challenged the Council to justify the ODA cuts that had been introduced under the current global conditions (EP resolution of 23 July). After long and protracted negotiations, the Parliament and the Council agreed on the final 2021–2027 budget framework, referred to as the EU’s Multiannual Financial Framework, which increased the aforementioned international cooperation budget to €79.5 billion (Keijzer, 2020).

In addition to process-oriented norms of “how much”, the EU put a strong focus on improving coordination, and the term “Team Europe” prominently featured in various EU public statements as well as in the observed online seminars about the EU’s global response (W2; W3). Accompanying press releases on Team Europe emphasized process-oriented EU development policy norms, most frequently by highlighting that Team Europe encompasses resources from the EU, its member states, the European Investment Bank, and the European Bank for Reconstruction and Development (W3).

The Team Europe “package” builds on and seeks to further promote the EU’s so-called working better together agenda. This agenda was the EU’s attempt to coordinate analysis and development cooperation planning decisions, and in the context of the pandemic, it focussed on ensuring that such joint strategies would address the pandemic response. Although the EU’s joint programming initiatives offer potential in this regard, so far joint programming has focussed on developing joint strategies rather than joined-up action. In addition, the EU’s joint programming approach faces a paradox between following developing-country leadership and promoting EU “self-coordination” (Carbone, 2017; Lundsgaarde and Keijzer, 2019). The EU’s 8 April Communication proposed to “reassign” planned EU aid expenditure to COVID-19-related purposes. The document was prepared without detailed consultations with developing countries, some of whom may have already budgeted this EU aid “income” in relation to other government spending priorities (EU, 2020b), thereby disregarding developing-country leadership (Jones et al., 2020).

Despite the frequent invocation of the term Team Europe in the policy proposals and press releases, it was not elaborated what Team Europe conveyed in operational terms. This deliberate ambiguity allowed for a high degree of flexibility and buy-in from EU member states. Instead of proposing new ways of working together, the EU’s approach in preparation of the 8 April Communication (EU, 2020b) mainly focussed on labelling and counting: The EU institutions took stock of their own and relevant member states’ contributions and presented the total overview as a “European approach”. Although this stock-taking exercise has its merits, it does not automatically create a European approach that is more than the sum of its parts. Furthermore, it gives the impression that the EU is most comfortable with its role in international development as the largest ODA provider (EU, 2020a). A truly joined-up approach would require concrete measures between the EU and its member states, ranging from health system strengthening initiatives in key affected developing countries to debt relief coordination.

In addition to debates on financing and Team Europe, EU development ministers also reaffirmed that a “stronger coordination between external and internal policies” is central to the implementation of the 2030 Agenda, the Paris Agreement, and the EU’s own long-term development policy. Public debates also emphasized the importance of coherence in internal–external action (W1). Since the start of the von der Leyen Commission, external action reforms and the development of a “geopolitical Commission” were meant to increase the Union’s effectiveness and visibility and to demonstrate “EU added value” in its pursuit of global goals. Consequently, Team Europe is following the same objective of highlighting the added value of a common European approach and the added visibility as a unified actor.

With the next seven-year budget having entered into force, further COVID-19-related cooperation is prepared through concrete “Team Europe initiatives” (TEIs). It remains to be seen to what extent these initiatives will contribute to changing the practice of cooperation across EU institutions and member states in the longer term. In most partner countries of the EU’s development cooperation, bottom-up preparations for the TEIs started in the summer of 2020, soon after the Team Europe Council Conclusions had been adopted. European Union Delegations on the ground consulted with EU member state embassies and other representatives as well as with partner-country officials. The Delegations subsequently submitted the proposed TEIs to Brussels for further discussions and consultations with the member states. Although proposed and shaped at the time to reflect that Team Europe was mainly associated with the EU’s response to COVID-19, these TEIs cover a longer period of cooperation and are much broader in terms of the cooperation substance they address. It is thus evident that all future development cooperation in the coming years will either directly or indirectly be affected by, or challenged to respond to, the consequences of the pandemic.

## Second priority: Multilateral solutions to global development

The transboundary nature of the pandemic has driven home the message that the crisis can only be tackled by a strong multilateral response and that the multilateral order needs to be reinforced to prevent further disruptions. As many countries chose a “my country first” approach and relations between China and the United States deteriorated, other international bodies and institutions that could have led a globally coordinated response—such as the G20 and the UN Security Council—were rather silent in the very early phase of the outbreak. The G20 took until the end of March to declare that the club was “committed to do whatever it takes to overcome the pandemic”, but plans for closer coordination on policy responses between its member countries remained vague. Due to disagreements between China and the United States, the UN Security Council took even longer and only discussed the pandemic on 9 April, failing to come up with a statement or resolution. Similarly, the US insistence on the virus being a “Chinese virus” made it difficult for the G7 to agree on a joint statement. This lack of global leadership opened the way for the EU to visibly act as a global leader.

The EU understands itself to be a leader in the coordinated global response—a role based on Article 21 of the TEU, which instructs the Union to promote multilateral solutions to common problems in the framework of the UN. Overall, in its official communication, the EU highlights the importance of relying on multilateral institutions to provide a global answer to the pandemic that builds upon past efforts. Illustrative of this is that the EU channelled a significant amount of funds to UN agencies and programmes, particularly UNITAID and the UN Development Programme (UNDP), in support of partner countries. Individual member states, such as Germany, also put a strong focus on multilateralism by supporting the coordinating role of WHO with other key organizations, such as the African Union (AU). In early April, the EU’s High Representative for Foreign Affairs and Security Policy, Josep Borrell, stated: “The coronavirus pandemic requires united, global action in response. The European Union and its Member States are playing their part in tackling this health crisis and its severe consequences—at home and abroad. (…) Cooperation and joint efforts at the international level and multilateral solutions are the way forward, for a true global agenda for the future” (EU, 2020f).

The need for a multilateral response to the crisis is coupled with the idea that the EU has a leading role in defending the multilateral liberal order. The EU Joint Communication of 8 April (EU, 2020b) is largely devoted to multilateralism, which is the most prominent principle emerging from the documents. It strongly emphasizes the need for global coordination to tackle the crisis and the relevance to rely on multilateral bodies, notably the G20, the G7, the UN, and multilateral financial institutions. The EU also commits to continuously work with the AU on the “Comprehensive Strategy with Africa”, for which it proposed key elements in March 2020. Although the summit eventually became a victim of the ongoing pandemic and was postponed first to 2021 and later to 2022, the EU’s strong commitment to this forum remained in place. The Commission announced several initiatives and funds that would be channelled through multilateral institutions, as illustrated by a €50 million contribution to UNDP to implement the UN Response Plan to COVID-19 in Nigeria.

As of 4 May, the EU stepped up and responded to WHO’s call for global action by organising a global pledging conference to raise funds from various sectors. Gradually, the EU assumed a leading role in coordinating the global action against COVID-19 in close cooperation with multilateral institutions, particularly in the search for a vaccine. As a distinguishing feature, and in contrast to other powers, the EU pledged to not only help find a vaccine, but also to make it affordable and universally available. This was one of the key fronts where the EU spoke with one voice and sent a message grounded on the principle of international solidarity.

The international pledging event was announced in a press release on 4 May, in which the EU stressed that “the Coronavirus Global Response builds on the commitment made by G20 leaders on 26 March”. Although it is a shorter document for the broader public, the text frequently alludes to the idea of multilateralism and the responsibility of the EU to assume a leading coordinating role in the global response.

The EU’s voiced intention to make a vaccine universally available constitutes more than a pledge of solidarity. It is equally a means to portray the EU as a normative power and an advocate of a global society in which solidarity prevails. As such, the EU’s commitment can be understood as a means of forging its identity while promoting a positive image of Europe abroad. References to the EU’s early material support to China and its proclamation to fight “any attempts of disinformation inside and outside the EU” as part of its global response to COVID-19 (EU, 2020b) are not incidental—they are proof of the geopolitical dimension linked to the Team Europe approach. In his personal blog, Borrell remarked on 23 March 2020 that “we must be aware there is a geo-political component including a struggle for influence through spinning and the ‘politics of generosity’” (Borrell 2020). The EU’s discreet support to China—on China’s explicit request—was answered with highly publicized and displayed Chinese support for Italy and Serbia (EU 2020d; Small 2020). These early incidents, as well as limited cases of actual competition between EU member states over access to materials, served as a wake-up call to EU leaders, sensitizing them to the pandemic’s geopolitical dimensions.

The EU’s role was put to the test when the United States announced their withdrawal from WHO at the end of May 2020. The EU’s response included a joint declaration expressing support for WHO “to continue being able to lead the international response to pandemics, current and future” before initiating a WHO resolution calling for an evaluation to review lessons learnt from the international health response to the coronavirus (EU 2020e). At other times, as participants in some of the online seminars observed, the EU displayed a more instrumental understanding of the role of multilateral organizations in delivering the global response to COVID-19, occasionally mentioning them in the same breath as the private sector (W3).

The Council conclusions on Team Europe adopted on 8 June further stressed “the need to support effective multilateralism and the importance of coordinating global action with the UN, regional organizations, notably the African Union, and other international multilateral organizations and financial institutions in the response to this global crisis” (EU 2020c). In addition, the Council welcomed what it judged as “comprehensive efforts made by the International Monetary Fund and the multilateral development banks to significantly accelerate their support to developing countries in their response to the crisis (…) and the initiative of the G20 and the Paris Club to provide debt service suspension for the poorest countries”. The Council further recognized the role of WHO to strengthen health systems and to prevent other pandemics. Another aspect that points to the EU’s adherence to multilateralism is the expressed reinforcement of European support for the Global Fund, GAVI, and UNITAID.

## Conclusion

This article analyzed the approaches and processes through which the EU articulates and diffuses its development policy norms. Its main aim was to determine to what extent the EU’s Team Europe response to COVID-19 articulated the Union’s overarching development policy norms, as enshrined in the EU’s treaties and policy statements. The two types of development policy norms, which are assumed to drive the engagement of the EU and its member states, can be grouped into process-oriented norms (emphasizing collective action) and substantive norms (emphasizing key values that the EU promotes).

Reflecting on the common adage that EU integration progresses in response to crises, the EU sought to use the COVID-19 pandemic to further the integration in the field of development policy. Team Europe’s proposed “leitmotiv” clearly accentuates the EU institutions’ desire to ensure that the Union and its member states provide a collective response that is greater than the sum of its individual parts. Our analysis finds that the Team Europe package matters greatly to further promote European integration in development policy and increase Europe’s profile and collective visibility as a development cooperation actor. The analysis of the two overarching priorities in its response, namely joined-up action and the promotion of multilateral solutions, demonstrates that the barriers to such further integration have not yet been overcome.

A second key conclusion of the paper is that, in the first half of 2020, the EU’s response greatly emphasized process-oriented norms over substantive development policy norms. Although the EU introduced important initiatives and actions, the overall picture remains that during the first wave of the pandemic, the EU and member states took individual actions while putting emphasis on strategically communicating these as a joint European response. It remains to be seen to what extent the implementation of the new Multiannual Financial Framework and the TEIs, as they have evolved since July 2020, will increase the EU’s capacity to move towards joined-up action.

In the longer run, EU member states will have to touch upon the critical question of whether they are willing to move towards deeper integration in European development policy and provide EU institutions with a stronger coordinating role in the field of development policy. The challenge to deliver a unified EU global response to the pandemic clearly reveals the intricacies of the nature of EU development policy as a shared competence between the EU and the bilateral development policies of its 27 member states. However, the realization that this institutional construction has its downsides in urgent crisis situations in which swift, joint action is needed may also create a window of opportunity to address the question of deeper integration.

Whereas this article has focussed on the EU’s articulation of its development policy norms in its proposed COVID-19 response in the first half of 2020, a key priority for future research should be the extent to which the policy choices were based on consultations with developing countries and reflected their needs and priorities. Given that several developing countries in the regions prioritized in the EU’s response remain ODA-dependent—especially several least developed countries in Africa—their public spending and planning may have been directly affected by the EU’s choices in terms of redirecting ODA, and would thus have warranted such consultations. The external perception of the EU’s development policy norms, both in this specific case and beyond, thus warrants further research inquiry.

## Annexure

See Annexure Tables 2 and 3.

**Table 2:** List of observed online seminars

W1: 7 May 2020, Webinar European Perspectives on the NEXUS at the Time of COVID-19 (Norwegian Refugee Council & Egmont Institute)
W2: 28 May 2020, The COVID-19 Pandemic Crisis and the Impact on Health Systems (European Commission)
W3: 10 June 2020, Team Europe Working Around the Clock: The EU Global Response to the COVID-19 (German Marshall Fund)
W4: 17 June 2020: Meeting of the European Parliament Development Committee on the EU’s Global Response to COVID-19 (European Parliament)
W5: 13. May 2020: Mit dem Green Deal aus der Krise? (MEP Delara Burkhardt, S&D party group)

**Table 3:** Official EU press releases and political statements. Source: Own elaboration

(1) EU press releases	(2) Political statements
8 April: “Coronavirus: European Union launches “Team Europe” package to support partner countries with more than €20 billion” 4 May: “Coronavirus global response: €7.4 billion raised for universal access to vaccines” 8 June: “‘Team Europe’ global response to COVID-19: Council welcomes the mobilization of almost €36 billion and approves conclusions”	16 April: “Only victory in Africa can end the pandemic everywhere”—joint op-ed by 18 EU and African leaders 8 June: Council conclusions on Team Europe global response to COVID-19

## Footnotes

1 Communications serve to capture and suggest key changes in EU development policy, and they play a key role in development policy due to the limited amount of “hard legislation” produced (Bergmann et al, 2019:539). The remainder of the article refers to Communication and occasionally to “proposal”.

2 Council Conclusions do not intend to have legal effects, but they are political statements that express the EU member states’ positions on a topic related to the EU’s areas of activity. The remainder of this article refers alternatively to Council Conclusions and a ministerial statement in relation to Team Europe.
